# Open-sourced modeling and simulating tools for decision-makers during an emerging pandemic or epidemic – Systematic evaluation of utility and usability: A scoping review update

**DOI:** 10.1016/j.dialog.2024.100189

**Published:** 2024-09-03

**Authors:** Rebecca Sophia Lais, Julia Fitzner, Yeon-Kyeng Lee, Verena Struckmann

**Affiliations:** a Charité - Universitätsmedizin Berlin, Charitéplatz 1, 10117 Berlin, Germany; b WHO Hub for Pandemic and Epidemic Intelligence Prinzessinnenstr, 17-18, 10969 Berlin, Germany; c Korea Disease Control and Prevention Agency, Osong Health Technology Administration Complex, 187, Osongsaengmyeong 2-ro, Osong-eup, Heungdeok-gu, Cheongju-si. Chungcheongbuk-do, Republic of Korea; d Berlin University of Technology, Department of Health Care Management, Straße des 17. Juni 135, 10623 Berlin, Germany

**Keywords:** Decision support, Modeling, Tool evaluation, Outbreak response, Pandemic, Epidemic

## Abstract

**Introduction:**

The COVID-19 pandemic had devastating effects on health systems globally. Emerging infectious diseases and pandemics will persist as a global health threat and preparedness for an evidence based response becomes challenging for decision makers. Epidemiological modeling can and has supported decision-making throughout pandemics. This study provides an update of the review “Publicly available software tools for decision-makers during an emergent epidemic—Systematic evaluation of utility and usability”^1.^

**Research question:**

What epidemiological modeling tools for decision-makers are open-sourced available for the usage in emerging epidemics or pandemics and how useful and user-friendly are these tools?

**Methods:**

A scoping review was conducted. We identified relevant studies through a search of peer-reviewed (Medline Ovid, Embase Ovid, PubMed, Cochrane) and gray literature databases, search engines such as Google, searches through stakeholder websites as well as expert consultations.

**Results:**

Of the 66 identified epidemiological modeling tools, 29 were included and qualitatively assessed using five-point-rating scales. The tools showed a good baseline of user-friendliness with variations in assessed components, features and utility. Room for improvement was found, specifically the capability to incorporate external data sources, detailed population descriptions, and geographic resolution.

**Discussion:**

Development efforts should prioritize clear communication of uncertainties and expert review processes. Trainings for specific tools should be considered.

**Conclusion:**

Tool usage can enhance decision-making when adapted to the user's needs and purpose. They should be consulted critically rather than followed blindly.

## 1 Introduction

With the onset of coronavirus disease 2019 (COVID-19), it again became evident that public health emergencies swiftly affect the global landscape. Considering that emerging infectious diseases will persist as a growing global threat [2], preparedness for evidence-based response, availability of user-friendly epidemiological tools, and the imperative of rapid decision-making in policy and healthcare are among the utmost priorities [3,4]. Rapid decision-making is challenging, as the choices must be well-informed, considering all relevant information and options at the same time. Meanwhile, infectious diseases transmission dynamics do not follow simple or always the same patterns, making decision-making very complicated [5]. As a result, expert opinions from just one field are not sufficient, instead several information sources and considerations must be considered to disentangle the underlying determinants in order to be able to make informed decisions [6].

Epidemiological modeling can and has supported complicated decision-making process throughout pandemics, such as the COVID-19 or influenza pandemic [7,8]. They enable the provision of quickly generated guidance [9,10] and consolidate scientific knowledge, which would be difficult or impossible to measure empirically [11,12], making the information-gathering process less complicated. Researchers trained in software languages and tools can program models based on different equations to simulate and analyze pandemic and epidemic dynamics [13]. However, the translation from science to decision is often lacking [6]. This step would benefit from computational tools to integrate and synthesize information faster [14,15].

Computational epidemiological modeling tools available for decision-makers can provide a scientific basis for policy and healthcare decision-making, allowing for the anticipation of disease spread and assessment of intervention strategies [16]. These tools can bridge the gap between complex scientific data and actionable insights, enabling better responses to public health emergencies [17] If utilized and crafted effectively, software tools tailored for modeling or simulation can aid decision-making processes, acting as supportive instruments or translators between academia and practical applications. While modeling involves creating a representation of a real-world system, simulation uses these models to study the system's behavior over time. These two concepts are not interchangeable, but they are often used together because models provide the framework for simulations to test hypotheses and gain insights into complex systems. Although not all modeling tools perform simulations, models are frequently used to simulate projections [18], which can greatly assist in decision-making processes.

This can be challenging as tools may be over-simplified or challenging to use [1]. Additionally, examining the framework of a model is crucial as it enables cross-checking results using consistent methods, ensuring that the model is used effectively with a clear understanding of its uncertainties [18]. This careful approach enhances the model's usefulness and reliability in decision-making [19].

To effectively utilize these tools, decision-makers must be aware of their existence and purpose, while accessibility, utility, and usability should be straightforward. To create an overview of these tools, Heslop et al. (2017) [1] conducted a review titled “Publicly Available Software Tools for Decision-makers during an Emergent Epidemic—Systematic Evaluation of Utility and Usability,” which evaluated usable tools up to July 2016. In this review, component measures such as e.g. flexibility, scalability, overall utility and usability were assessed, focusing on tools helpful in emergent epidemics. An overview describing the tools characteristics was created and overall results compared. Given the heightened reliance on modeling tools during the COVID-19 pandemic [20], new modeling tools will likely be developed, and existing ones will probably be updated. This increases the likelihood that new evidence has recently emerged, suggesting that an update to the review would be beneficial [21].

Therefore, this study aims to map existing tools, provide an updated analysis of the previous review conducted by Heslop et al. [1], and explore the scope and characteristics of modeling tools that have become publicly available since July 2016. This review will examine tools' components, features, and limitations while evaluating their utility and usability to provide a qualitatively analyzed overview of currently developed tools for decision-makers, similarly to Heslop et al.

Additionally, minor methodological and content changes were performed, which may be warranted in an updated review [22]. This review transitioned from a systematic review conducted by Heslop et al. to a scoping review. The reason for this was, that the aim of this review was to map the increasing breath of evidence available and to report on the characteristics of identified diverse tools. These criteria align with those used by JBI to determine when a scoping review is appropriate [23]. Additionally, a methodological quality assessment of the publications in which the tools are identified, are not relevant for the outcome of this study, as the purpose is to evaluate the tools, not the publications. This also speaks for conducting a scoping review not a systematic review [24]. A further adaption is that this research extends beyond tools exclusively designed for epidemics and also includes tools developed for pandemics and an adaptation of the usability and utility rating was conducted. As the review by Heslop et al., rated overall usability and utility, this review created a more detailed rating scheme also for these characteristics. Additionally, component measures were added.

## 2 Material and methods

To conduct this scoping review, an extensive literature search was performed between August and November 2023, following the guidelines of the updated methodological guidance for conducting scoping reviews within the Joana Briggs Institute Evidence Implementation [25]. This search included peer-reviewed databases, internet search engines, stakeholder websites, and a Korea Disease Control and Prevention Agency (KDCA) expert consultation. The expert consultation with [YKL] was conducted via a videoconference in October, resulting in a list in an MS Excel® file format containing epidemiological modeling tools. This list can be accessed via the A1 File. Search results were qualitatively assessed and reported using the Checklist created in the PRISMA extension for Scoping Reviews [26]. The review is registered with the registration number 62J5Z via the Open Science Framework, and the protocol is attached in the A2 File. A comprehensive search string was created jointly by all authors with the support of a librarian from each, the Charité and the World Health Organization (WHO) library to identify English language articles published since 2016. Search options, especially online search engines, gray literature, and stakeholder databases, are less advanced than those in peer-reviewed databases. Hence, more derivation can be found in these strings. The different search strings can be found in the A3 File.

### 2.1 Database

Medline Ovid, Embase Ovid, PubMed, and Cochrane were searched for peer-reviewed articles. After the search of peer-reviewed databases, hits were exported and uploaded to Rayyan. First the titles and abstracts of the identified papers were screened for relevance by two independent reviewers (YL and RL) in blind mode. Rayyan's AI deduplication was used and double-checked by one reviewer (RL). Publications considered relevant only by one of the two reviewers were discussed until consensus was reached or any differences were resolved by a third researcher (SKS), then the full text publication was retrieved.

Base and OpenGrey were used for gray literature, the Institutional Repository for Information Sharing of WHO (IRIS WHO), and the Centers for Disease Control and Prevention (CDC) Website for stakeholder websites. Hits were copied into a MS Excel® sheet. Two independent reviewers (YL and RL) used separate sheets for screening titles and abstracts. Deduplication was performed using Microsoft (MS) Excel®; one reviewer (RL) controlled the deduplication process. Any differences were resolved by a third researcher (SKS). Google, Yahoo, and Bing were searched for internet search engines in incognito mode. Since advanced search options and Boolean operators are not functional in Google, Yahoo, and Bing, four keyword searches were conducted using the search string's terms for each search engine. The first hundred results of each keyword search, hence 400 hits per search engine, were screened. Results were copied into an MS Excel® sheet for documentation. This enabled two reviewers (YL and RL) to screen title and abstract of the results without receiving personalized hits in the search engine. Deduplication was carried out in the same way as the gray literature with conflicts reviewed by a third researcher (SKS) and resolved based on majority agreement.

Ten percent of all the full-text articles (*n* = 25) were screened by two researchers (YL and RL) and a low level of disagreement (*n* = 3) was identified. These two reviewers independently piloted the in- and exclusion criteria, followed by full-text article screening of the remaining by one reviewer (RL).

### 2.2 Article selection

**Fig. 1:**
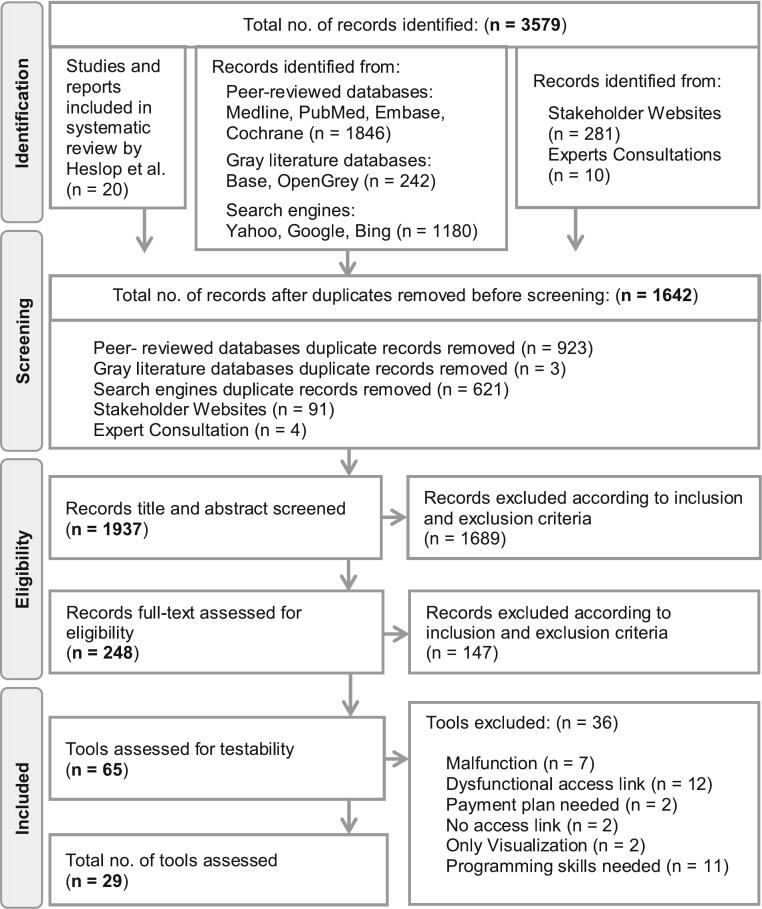
Flow chart.

Each article was screened based on a pre-defined set of inclusion and exclusion criteria, these criteria can be found in the A6 file. The article selection process is illustrated in Fig. 1. One researcher (RL) developed the criteria, followed by a second researcher (YL) to prove understanding. The final set of inclusion and exclusion criteria were checked and adapted by a third researcher (VS).

Papers and articles describing open-sourced or openly available epidemiological modeling or simulation tools for infectious diseases with pandemic or epidemic potential were included. Tools assessed by Heslop et al. [1] were re-evaluated. Systematic reviews and tools that required a payment for full access and use were excluded. Tools that were not testable due to installation problems, malfunction, or lacking legal copyright were also excluded. Tools published before July 2016 and those not written in English or German were excluded. The target group of this review are decision-makers, it cannot be assumed that they have coding skills, so tools with the requirement for additional software like “R” or advanced coding knowledge were excluded. In this review, the term “decision-makers” encompasses both individuals in management roles, such as hospital managers, leaders of community organizations, and business executives, and those involved in policy-making at various governmental tiers, including the national, provincial, district, municipal and local levels [27].

### 2.3 Data extraction and qualitative assessment

The qualitative assessment was developed based on rating criteria and a 5-point rating scale created by Heslop et al. (2017). It then underwent an extension and adaptation based on the input of experts, including WHO epidemiologists, a WHO UI and UX expert, and a research fellow at the Technical University Berlin. Functionality features, component measures, and utility and usability of the tools were assessed. Elements of the Health IT Usability Evaluation Scale [28] and a website usability checklist [29] were also incorporated to further strengthen the existing assessment criteria. Then, a standardized rating scheme was created in MS Excel® to assess each tool. The 5-point scale descriptions used for the component measures can be found in Table 1.

**Table 1:** 5-point scale description for component measures.

Component Measures - 5-point rating scale					
Rating	5	4	3	2	1
Flexibility	Complete flexibility in selecting parametri-zation formats and types and data.	Moderate flexibility in selecting parametri-zation formats and types and data.	Some flexibility in selecting parametri-zation formats and types and data.	Limited/little flexibility in selecting parametri-zation formats and types and data.	No flexibility in selecting parametri-zations formats and types and data.
Adaptability	Adaptable to any context and any situation.	Can be adapted to pathogens across classes, or species, in many contexts.	Can be adapted to pathogens in one class only, in limited contexts.	Can be adapted to one or a narrow range of pathogens in one context only.	One pathogen, in one context only.
Scalability	User defined scaling to any population or parameter size.	Scaling allowed within assumptions of the modeling technique.	Scaling within some contexts, according to rules or constraints.	Can scale within narrow limits or in narrow context.	No scalability features.
Geographic Resolution	User defined, without pre-set limitation, up to and including individual level GIS continuous.	GIS with defined areas (e.g. census district).	Sub jurisdictional divisions allowed or supported.	Multi-area or multi-compartment.	Single jurisdiction or compartment.
Time Resolution	Minutes	Days	Weeks	Months	Years
Population Description	User can adapt several population descriptors. Easily handles many different types of populations & is very flexible.	User can adapt a choice of differences in popu-lations, providing a good understanding of influencing parameters. Handles a wide range of different populations.	User can adapt some population differences. Handles some differences in popu-lations; might struggle with others.	Population differences can be adapted by the user, but it's basic. Can't easily handle different types of populations.	Doesn't allow the adaptation of popu-lation differences. Not flexible and can't handle different types of populations well.
Computer Platform Compatibility	Any	3 Platforms supported, Plus Mobile Devices.	3 Platforms supported.	2 Platforms supported.	1 Platform supported.
Transparency	Excels in sharing results, provides comprehensive details about calculations in a highly user-friendly and efficient way.	Allows for sharing results, and it provides adequate details about the calculations in a user-friendly manner.	Some capability to share results and calculation details, but it's neither remarkable nor problematic.	Sharing results or calculation details is possible, but it's not user-friendly or convenient.	Does not provide any option to share results or details about calculations after usage.
Reproduc- ability	Excels in reproducibility, providing robust features for saving calculations and easy re-executions, contributing to a highly reproducible workflow.	Supports reproducibility well, allowing users to save calculations and efficiently re-run them at a later stage.	Offers basic features for saving and re-running calculations, but it could be improved for better reproducibility.	Reproducibility features are limited; saving and re-running calculations are possible but not user-friendly.	Does not allow saving calculations or re-running them at a later stage, hindering reproducibility.
Data Linkage	Set of given data is linkable. Tool automatic-ally updates data set using real-time data. Data sets are exchange-able as per disease.	A set of given data is linkable to the tool. Data is exchangeable as per disease.	A set of given data is linkable to the tool.	Data is linkable within narrow limits.	There is no new linkage to data or it cannot be found, if the data set is adaptable.
Versatility	Contains all of the listed features.	Contains 12–16 of the listed features.	Contains 8–12 of the listed features.	Contains 4–8 of the listed features.	Contains <4 of the listed features.
Visual-ization	Allows a variety of visualizations, which can be adapted in language, show observational & analytical visuals & can be exported in different formats.	Allows a small choice of visualizations, which can be exported in different formats, but cannot change the language within visuals.	Allows a a small choice of visualizations, which can not be adapted in language and cannot be exported in different formats.	Allows more than one way of visualization, which can not be adapted in either language or format.	Allows one simple, not adaptable visualization of the results.

The utility and usability rating scale is documented in Table 2.

**Table 2:** 5-point scale description for utility & usability.

	Utility & Usability - 5-point rating scale					
	Rating	5	4	3	2	1
Utility	General Usefulness	Highly useful for supporting decision-making. Provides excellent features & functionality for effective decision support.	Generally useful for supporting decision-making, there may be some areas for improvement.	Has both positive & negative aspects for supporting decision-making. Its usefulness is neither strongly affirmed nor denied.	Has some limitations in supporting decision-making. Its usefulness is questionable.	Does not support decision making. Lacks the necessary features or functionality.
	Process usefulness	Creates a very transparent way of making decisions. No improvements are needed.	Can create a more transparent way of making decisions, for decisions within the set framework of the tool. Some aspects are unclear. Improvements would enhance understanding.	Can create a more transparent way of making decisions, for decisions within specific contexts. It's neither notably lacking nor outst&ing. There may be room for improvement.	Can create a more transparent way of making decisions, for decisions within a limited context. It is generally sufficient, with room for improvement.	Does not create a more transparent way of decision-making. Significant improvements needed to make the decision-making process more clear & understandable.
	Results usefulness	Produces results for decision-making within any population, parameter size, inter-ventions. Results significantly enhance the decision-making process by providing clear & valuable direction.	Produces results for desicion-making within the assumptions of the modeling technique used. There may be areas for improvement, but overall, the results contribute positively to the decision-making process.	Produces results for decision-making within some contexts, according to rules or constraints. It could be more explicit or comprehensive.	Produces results for decision making within narrow limits or in narrow context. Improvements are needed in offering clear direction.	Does not produce results, which are helpful for decision making processes. It lacks support or direction in this regard.
Usability	Learnability	Really easy to learn how to operate the tool. User manual is rarely needed.	It is easy to learn how to operate the tool using the user manual.	Learning how to operate the tool is not outst&ingly easy nor complex. It works with the user manual.	Learning how to operate the tool is complex. The user manual is incomplete & not very helpful.	Learning how to operate the tool is very complex. There is no user manual.
	Ease of use	Very easy to use. It works intuitively explanations are rarely needed.	Easy to use.	Usage is not too complex, but sometimes needs further explanations to use alll functions properly.	Usage is complex. User manual is incomplete & not very helpful.	Usage is very complex. There is no user manual.
	Memor- ability	Very easy to remember how to log on to the tool & how to use it, even after not using it for a longer period.	Easy to remember how to log on to the tool & how to use it.	More complex to remember how to log on to the tool & how to use it, but its understandable after the first usage.	Difficult to remember how to log on to the tool & how to use it. It gets easier the more frequent the tool is used.	Really difficult remember how to log on to the tool & how to use it, already after using it frequently.
	Error Prevention	Gives very clear & easy-to-understand messages, telling the user how to fix problems when they occur. The solutions they provide are resolving the problems in an easy way.	Gives mainly clear & easy-to-understand messages, telling the user how to fix problems when they occur. The solutions the provide are mostly resolving the problems in an underst&able way.	Gives error messages once problems occur. The solutions they provide are resolving the problems in a rather complex way.	Sometimes gives error messages once problems occur. The solutions they provide are not resolving the problems.	Does not give error messages once problems occur.
	Information Needs	Information is exceptionally clear, making it easy for users to grasp the tool's functionalities. On-screen messages, online help, & documentation are well-organized, concise, & cater to the diverse needs of users.	Information is generally clear, providing users with a good understanding of the tool through on-screen messages, online help, & documentation. Users can navigate & use the tool without significant confusion.	Information provided is clear to a moderate extent. While some users may find it sufficient, others might face challenges in under-standing certain aspects. There is room for improvement to ensure widespread clarity.	Information is somewhat unclear, requiring users to put in extra effort to comprehend on-screen messages & documentation. Improvement is needed to enhance user understanding.	Information is highly unclear, making it difficult for users to understand how to use the tool effectively. On-screen messages, online help, & documentation are confusing or not available & lack coherence.
	Feedback	Shows interaction feedback in every neccessary function in an easy to understand manner.	Shows interaction feedback, which is mostly easy to understand. There is room for improvement.	Shows interaction feedback when needed. The meaning is not very easy to understand.	Shows interaction feedback, but not in every neccessary function. The meaning is not understandable.	Does not show any interaction feedback.
	Navigation	Navigation options in the menu can be easily found & are always clear & visible.	Navigation options in the menu can be found & are mostly clear & visible.	Navigation options can be found but are not very clear.	Navigation options in the menu can be found. They are not clear & visible.	Navigation options in the menu cannot be found & hence are not clear or visible.
	Intuitive Programm- ing	Topics are consistently & logically grouped, providing a highly intuitive user experience.	Related topics are generally grouped together, contributing to an intuitive programming experience.	The organization of topics is neither particularly intuitive nor confusing; it's average.	Some related topics are grouped, but the organization is not intuitive, causing confusion.	Topics are scattered & not logically grouped, making it difficult to find related information.

Additionally, essential tool characteristics were documented. These characteristics are collated in Table 4, while the access links are in the A4 File. Furthermore, desirable features of the tools were listed following a binary checklist, indicating whether the tested tools included those features. Table 3 overviews the considered features followed by their definition.

**Table 3:** Definition of functionality features.

Functionality Features	
Features	Definitions
Transport Modeling	Able to model movement of individuals between geographic areas.
Demographic Modeling	Able to incorporate divisions of population based on age and other demographic features.
Complex Systems Features	Able to incorporate complex adaptive system modeling, such as feedback, agent-environment interaction or recursive modeling.
HPC Support	Can utilize High Performance Computing (i.e. supercomputing) to enable larger scale or more detailed modeling tasks.
Real Time Capabilities	The end user can employ the tool to support decision making in real-time, within minutes to hours.
Retrospective Analysis	The tool supports entry of data that provides the ability to retrospectively analyze an historical event.
Prospective Analysis	The tool generates snapshot or time-series data that allows for prediction of features of an epidemic based on input parameters and assumptions.
Decision Support	The tool is designed to directly support decision making, or addresses a specific question related to epidemic response decision making.
Risk Management Support	The tool is designed to generate data that supports the development of risk analyses directly.
Machine Learning Support	The tool has features that allow the system to adapt to previous scenarios or results, potentially without user direction or input.
GIS Support Capabilities	The tool has features that allow Geospatial Information Support
Multimodal user support	The tool has features that allow users to interact withh a system through supported modalities.
Parameter adjustment	The tool allows to adjust parameters.
Data set upload	The tool allowes to upload predefined datasets (e.g in an excel sheet).
Automated data update	The tool is able to use real time data.
Disease specific data changes	The tool is able to adapt data sets and interventions depending on the disease.
Exportoptions	The tool is able to export results in at least one other data format to allow continuing work with other programs (e.g excel or RIS).
Code is adaptable	The tool allows the adaptation of the code.
Cost-effectiveness	The tool calculates costs of the interventions.

Two researchers (YL and RL) independently tested each tool using the rating scale (Table 1). Results were collated in two separate MS Excel® documents. Rating differences were assessed by a third researcher (JF). As the tools are evaluated for use by decision-makers, an expert consultation with a decision-maker within the German Federal Ministry of Health for the best-rated 10 % of the tools was conducted. For this purpose, the questions were reduced to the utility and usability criteria, being the most relevant from a decision-makers' perspective.

## 3 Results

After applying the pre-defined criteria, 46 tools developed after July 2016 were identified in all databases. Adding the 20 previously examined by Heslop et al. (2017), 66 tools were assessed further. Most tools were sourced from peer-reviewed databases and expert consultation, while a few additional were discovered through search engines. Gray literature search yielded only one additional relevant tool. A detailed flow chart outlining the search process is presented in Fig. 1.

Six of the twenty tools evaluated by Heslop et al. were still online, updated since the review and included in the assessment. Twenty-three tools fitting the inclusion criteria, developed after July 2016 and available to test, underwent a complete evaluation. Thirty-six tools were excluded due to malfunction of the tool (19 %), dysfunctional access links (33 %), payment plan or access codes needed for the full scope (6 %), no access link available (6 %), only visualization tool (6 %) or required programming skills (31 %). A list of all excluded tools with reason for exclusion including the hyperlinks, can be found in the S5 File. Table 4 provides an overview with further information on the tested tools.

**Table 4:** Overview of evaluated modeling tools.

Description of evaluated modeling tools							
Tool Name	Tool description	Modeling Methodology	Pathogen	Data Input Requirements	Design	Updated	Created
A dynamic modeling tool	Evaluates intervention strategies, estimates healthcare demand during the COVID-19 epidemic, & assesses population-wide interventions.	D; Compartmental, SEIR	Covid	Defaults provided. Advanced use requires familiarity with & user input of numerical disease parameters & assumptions. Direct input of model parameters into custom program interface.	MS Excel®	–	2020
Anthrax Assist	Quickly forecasting the incidence of IA cases & providing decision support for response during an aerosolized anthrax event.	S; Wilkening model, Pep Impact Model, Compartemental	Inhalation anthrax	Direct input of model parameters into custom program interface, Defaults provided. Advanced use requires familiarity with & user input of numerical disease parameters & assumptions.	MS Excel®	2021	2017
BERM Version 2	Anticipates quantity & categories of personnel required to address a disease outbreak or bioterrorism incident within a specific population.	S; probabilistic state transition model, logistic model, prospective time series	Any	Web based entry of estimated model parameters & probabilities, with some default values provided.	Web-based	2005	2003
CEAT	Measures exposure by considering airflow, group behavior, & infection prevalence in the community.	S; wells-riley, Poisson probability distribution	Covid	Defaults provided. Advanced use requires familiarity with & user input of numerical disease parameters & assumptions.	Pdf-document	–	2022
CHIME	A modified SIR model for short-term outbreak forecasting.	D; Compartmental, SIR, Sigmoid function	Covid	Web based entry of estimated model parameters & probabilities, with some default values provided.	Web-based	2020	2020
Coronavirus Policy Response Simulator	Predicts the health & economic impacts at the state level when businesses reopen & stay-at-home orders are eased.	D; Compartmental, SIR+	Covid	Web based entry of estimated model parameters & probabilities, with some default values provided.	Web-based	–	2020
Covid Web app	Evaluate the effects of mitigating measures against COVID-19.	S&D, Compartmental	Covid	Direct input of model parameters into custom program interface, Defaults provided. Advanced use requires familiarity with & user input of numerical disease parameters & assumptions.	Web-based	–	2020
Covidscreen	Provide estimates & visual aids to assess organizational infection risk & testing benefits for informed decision-making.	S; Probability model	Covid	Web based entry of estimated model parameters & probabilities, with some default values provided.	Web-based	–	2022
COVIDTracer	Enabling state & local public health officials to compare three user-defined contact tracing & monitoring strategies, assessing effectiveness & resource requirements.	D; Compartemental	Covid	Direct input of model parameters into custom program interface, Defaults provided.	MS Excel®	2020	2020
Drive Through Mass Vaccination Sim for COVID19	Aid vaccination planners in evaluating outcomes for various drive-through vaccination facilities.	S; Discrete event, monte carlo	Covid	Web based entry of estimated model parameters & probabilities, with some default values provided.	Web-based	–	2020
GER⁎	Estimate the count of EVD cases in a community, monitor patients' susceptibility across infectivity, incubation, recovery, & death, & determine the spread & impact of EVD over a 300-day period.	D; Logistic requirements model, prospective snapshot	Ebola Virus Disease (EVD)	Defaults provided. Advanced use requires familiarity with & user input of numerical disease parameters & assumptions, Direct input of model parameters into custom program interface.	MS Excel®	New version, date is unclear.	2014
GleamViz ABM⁎	Facilitates policy-making & emergency planning by creating epidemic models & conducting scenario analyses to assess the real threat posed.	S; Compartmental	Any	Direct input of model parameters into custom program interface, Customised compartment models & transitions programmable using graphical interface.	Software	New version, date is unclear.	2011
Mriids	Real-time epidemic forecasting tool	S; Mathematical	Covid, Ebola	Web based entry of estimated model parameters & probabilities, with some default values provided.	Web-based	Data daily	2021
COVID-19Surge	To estimate the surge in demand for hospital services amid the COVID-19 pandemic.	D; Compartemental, SIICR	Covid	Direct input of model parameters into custom program interface, Defaults provided. Advanced use requires familiarity with & user input of numerical disease parameters & assumptions; Customised compartment models & transitions programmable using graphical interface.	MS Excel®	2023	2020

⁎ Tools evaluated by Heslop et al. 2017 | S = Stochastic | D = Deterministic.

Table 5 displays the qualitative assessments, with the results presented in descending order, with the highest average score on the left and the lowest on the right.

**Table 5:** Results of the qualitative assessment.

Results of the qualitative tool assessment																														
	Tool Name	Epidemix	CovidSim	Covid-19Surge	Covidscreen	A dynamic modeling tool	COVID Tracer	BERM	CHIME	MRIIDS	Anthrax Assist	Covid Webapp	GER*	Gleam Viz*	Coronavirus Policy Response Simulator	CEAT	Drive Through Mass Vaccination Sim	Epipop	BCoDE	Covid19Vaxplorer	PHL Impact tool	VacStockPile*	Netlogo Epidem	Tabby2	SISpread*	CPID*	Modeling COVID-19	Epilocal	Texas Pandemic*	CoSim
	Modeling methodolgy	S,D	D	D	S	D	D	S	D	S	S	S,D	D	S	D	S	S	S	S	D	n.a	D	D	S	D	D	D	D	D	D
1: Component Measures	Flexibility	4	5	4	3	3	4	3	2	2	3	5	4	3	2	4	4	4	4	3	2	2	2	4	3	2	2	3	3	3
	Adaptability	5	3	2	3	1	3	3	1	2	1	3	1	4	2	3	1	4	4	3	1	2	3	1	5	1	2	1	1	3
	Scalability	5	5	5	4	3	5	5	3	1	2	5	5	4	2	2	5	5	4	4	3	4	4	5	5	1	5	3	1	1
	Geographic resolution	3	4	1	1	1	1	1	1	2	1	5	1	2	2	1	1	1	2	2	1	1	1	3	5	2	1	2	4	4
	Time resolution	4	4	4	4	5	4	5	4	4	1	4	4	4	3	4	5	4	1	4	1	1	5	1	5	3	4	4	4	1
	Population description	4	4	1	5	3	1	2	1	1	1	5	2	1	2	2	1	1	3	2	1	1	1	1	1	1	2	2	3	1
	Computer platform compatibility	3	5	5	5	1	5	5	5	4	5	5	1	3	5	5	5	5	1	5	3	3	2	5	2	5	3	5	5	5
	Transparency	3	3	2	1	3	3	2	3	2	3	4	4	4	2	4	3	4	2	2	4	3	3	3	2	2	3	1	2	3
	Reproducability	2	5	4	1	5	4	1	4	1	4	4	4	5	1	4	1	5	1	3	4	4	4	1	1	1	4	1	1	1
	Data linkage	4	1	3	1	2	1	1	1	1	1	1	2	1	1	1	1	1	3	1	2	2	1	1	1	1	2	1	2	1
	Versatility	4	3	3	1	3	2	2	2	3	2	2	3	4	2	2	2	3	3	2	3	3	2	2	2	2	3	1	3	3
	Visualization	3	3	3	2	3	3	2	3	2	2	1	3	3	2	1	2	1	1	2	4	4	4	1	1	2	2	2	3	1
	**Total** of max. 60 pts.	44	45	37	31	33	36	32	30	25	26	44	34	38	26	33	31	38	29	33	29	30	32	28	33	23	33	26	32	27
2: Utility	General usefulness	4	4	4	5	4	3	4	4	4	4	3	2	4	2	3	3	2	4	4	3	4	2	3	1	2	2	3	3	2
	Process usefulness	4	3	3	4	3	3	3	3	2	4	3	1	3	2	2	2	1	3	3	3	2	2	2	1	1	2	2	1	1
	Results usefulness	3	4	4	4	3	4	3	3	2	3	4	2	3	2	2	2	2	3	2	3	3	2	3	2	1	2	1	1	2
	**Total** of max. 15 pts.	11	11	11	13	10	10	10	10	8	11	10	5	10	6	7	7	5	10	9	9	9	6	8	4	4	6	6	5	5
3: Usability	Learnability	5	5	5	5	5	5	4	5	5	5	2	5	3	5	4	5	4	4	4	5	4	4	5	3	4	4	4	3	5
	Ease of use	5	5	5	5	5	4	5	5	5	5	2	4	3	5	3	4	3	4	3	5	4	4	5	4	4	4	4	5	5
	Momorability	5	5	5	5	5	5	5	5	5	5	2	5	3	5	3	5	3	5	5	5	5	5	5	3	5	5	5	2	5
	Error prevention	5	1	4	1	1	2	4	2	5	2	1	1	2	5	4	5	3	1	3	2	2	1	1	2	5	1	2	1	1
	Information needs	5	5	5	5	5	3	3	4	4	5	4	5	2	2	3	3	3	4	2	4	3	3	3	3	4	1	2	2	1
	Feedback	4	4	4	5	1	2	2	2	5	2	1	2	2	5	3	3	2	2	2	1	2	2	1	2	2	2	2	3	2
	Navigation	5	5	4	5	5	4	4	5	5	5	3	5	4	5	4	3	4	4	4	4	4	4	5	4	5	2	4	3	1
	Intuitive programming	5	5	5	5	5	3	4	5	5	5	2	5	4	5	5	3	3	5	3	4	4	4	4	5	5	3	4	1	1
	Total of max. 40 pts.	39	35	37	36	32	28	31	33	39	34	17	32	23	37	29	31	25	29	26	30	28	27	29	26	34	22	27	20	21
1,2,3	**Overall**	**94**	**91**	**85**	**80**	**75**	**74**	**73**	**73**	**72**	**71**	**71**	**71**	**71**	**69**	**69**	**69**	**68**	**68**	**68**	**68**	**67**	**65**	**65**	**63**	**61**	**61**	**59**	**57**	**53**
	**Overall in %**	**82**	**79**	**74**	**70**	**65**	**64**	**63**	**63**	**63**	**62**	**62**	**62**	**62**	**60**	**60**	**60**	**59**	**59**	**59**	**59**	**58**	**57**	**57**	**55**	**53**	**53**	**51**	**50**	**46**
4: Functionality Features	Transport modeling	x												x				x												
	Demographic modeling		x			x		x					x						x					x					x	
	Complex systems features													x											x					
	HPC support													x															x	
	Real time capabilities	x	x	x		x	x	x	x	x	x		x	x	x	x	x	x	x	x	x	x	x		x	x	x	x	x	x
	Retrospective analysis		x	x			x			x		x		x	x	x		x			x					x	x	x		
	Prospective analysis	x	x	x		x	x	x	x	x	x	x	x	x	x	x	x	x	x	x		x	x	x	x	x	x		x	x
	Decision support	x	x	x	x	x	x	x	x	x	x		x	x	x	x	x	x	x	x	x	x	x		x	x	x		x	x
	Risk management support	x		x	x	x			x	x		x		x		x							x	x						x
	Machine learning support											x																		
	GIS support capabilities	x												x						x				x					x	
	Multimodal user support		x																		x				x					
	Parameter adjustment	x	x	x	x	x	x	x	x	x	x	x	x	x		x	x	x	x	x	x	x	x	x	x		x		x	x
	Data set upload	x	x	x								x	x					x	x			x					x			
	Automated data update									x		x									x							x		
	Disease specific data changes	x																x	x			x								
	Export options	x		x		x	x		x		x		x	x			x	x			x	x	x	x	x	x	x		x	x
	Code adaptability			x		x			x		x		x	x				x	x	x	x	x	x				x			
	Cost-effectiveness																				x	x		x						
	**Total no. of features**	**10**	**8**	**9**	**3**	**8**	**6**	**5**	**7**	**7**	**6**	**7**	**8**	**12**	**4**	**6**	**5**	**10**	**8**	**6**	**9**	**9**	**7**	**7**	**7**	**5**	**8**	**3**	**8**	**6**

S = Stochastic; D=Deterministic; * = re-evaluated tool, developed before 2016.

Deterministic models, like those used in large-scale disease spread predictions (e.g., the SIR model), provide consistent outcomes based on fixed parameters. In contrast, stochastic models, often applied in small populations to account for random events (e.g., disease spread in a small community), produce varying results that capture the system's inherent unpredictability [30]. Out of twenty-nine tools, sixteen were exclusively or predominantly deterministic, twelve were primarily stochastic, and one was unclear. The utilized models varied in thirteen of the tools, but sixteen used compartmental models. Among twenty-nine analyzed tools, fourteen were designed for purely COVID-19 modeling, eight for any pathogens, two for influenza, four for other specific pathogens, and two for Ebola of which one also allowed modeling for COVID-19. Among COVID-19 modeling tools, stochastic modeling methods are slightly more prevalent.

The overall qualitative assessment data reveals a comprehensive range in rating scores of epidemiological modeling tools, with scores from 46 % to 82 %. Epidemix is the highest-ranked tool with an overall score of 82 %, indicating superior performance across various metrics. In contrast, CoSim is at the lower end of the spectrum with a score of 46 %. Most tools scored within the mid to high range, showing a qualitative baseline functionality.

### 3.1 Component measures

Evaluated tools showed high computer platform compatibility and scalability performance, enabling accessibility across several computing environments and modeling at various scales. Most tools also displayed good ratings in time resolution, primarily operating on daily time intervals, enhancing the granularity of disease spread tracking and modeling. High reproducibility rates could be identified in most tools, which enables the tools to save calculations for later re-execution. In contrast, lower ratings were observed in data linkage, population description, versatility, geographic resolution, and visualization. Shortcomings were noted in effectively incorporating external data sources and detailed demographic modeling. While visualization outputs were prevalent among the tools, few offered flexibility in downloading visuals in diverse formats or adapting language settings. Versatility, describing the number of incorporated features, will be elaborated upon in the section on feature assessment 3.3. Transparency and flexibility received mainly moderate scores. The highest score across component measures had CovidSim, notably excelling in flexibility, scalability, and computer platform compatibility.

### 3.2 Usability and utility

Looking at the median, web apps achieved the highest usability ratings, followed by MS Excel® tools, PDF files, and software applications. Despite the variation in design, all tools shared similar lower scores for error feedback and information needs while achieving higher scores for navigation and intuitive design. MS Excel® tools and web services outperformed standalone PC applications regarding learnability, ease of use, and memorability. The utility ratings displayed a wide range, with the lowest-rated tool scoring four out of fifteen points and the highest-scoring thirteen out of fifteen points. Covidscreen, a web service, was the highest-rated tool in terms of utility, whereas the lowest-rated tools were SISspread and CPID, a software application and a web service. Both tools were developed before 2016.

During the expert consultation on utility and usability with a decision-maker from the Federal Ministry of Health, Germany, a preference for web apps was expressed due to the requirement for software to undergo stringent security checks, which complicates its usage. Furthermore, it was highlighted that using the tools effectively from the outset is challenging without regular engagement, as time is required to understand them fully.

### 3.3 Feature assessment

**Fig. 2:**
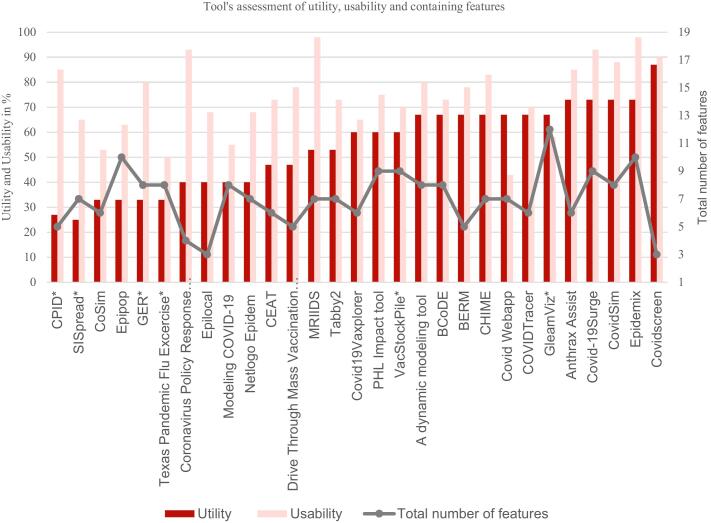
Assessment of utility, usability, and containing features.

Versatility of the tools was assessed by counting the numbers of available functionality features in each tool. Of the nineteen features assessed (see Table 5, point 4, functionality features), four were present in most of the tools tested. Namely, decision support, real-time capability, prospective analysis, and parameter adjustment. Fig. 2 provides an overview of each tool's features, compared to their utility and usability. This comparison shows that many features, hence a high versatility, are not necessary to make a tool useful, as seen with Covidscreen. At the same time, having many features does not automatically make a tool much less user-friendly, as Epipop and GleamViz show.

Decision support was offered by twenty-seven out of twenty-nine tools, meaning these tools could directly assist in decision-making within the design and assumptions of the modeling technique. With real-time capability in twenty-eight of twenty-nine tools, decision support could provide results through prospective analysis within minutes to hours. Prospective analysis predicts based on the input assumptions and parameters.

Parameter adjustment is essential to make a more accurate prediction. This feature was incorporated into twenty-seven tools. The extent of parameter adjustment varied significantly, with some tools allowing a complete parameter set upload while others allowed the adaptation of a minimal parameter set.

### 3.4 Usage conditions

All tested tools were openly available. They provided user manuals, available either through on-screen guides embedded in the tool, as downloadable PDF documents, or within a separate window in the web application. Although the details and ease of learnability varied between the tools, none scored lower than three. Hence, they were all moderately easy to learn.

### 3.5 Comparison tools rated by Heslop et al. and new tools

**Fig. 3:**
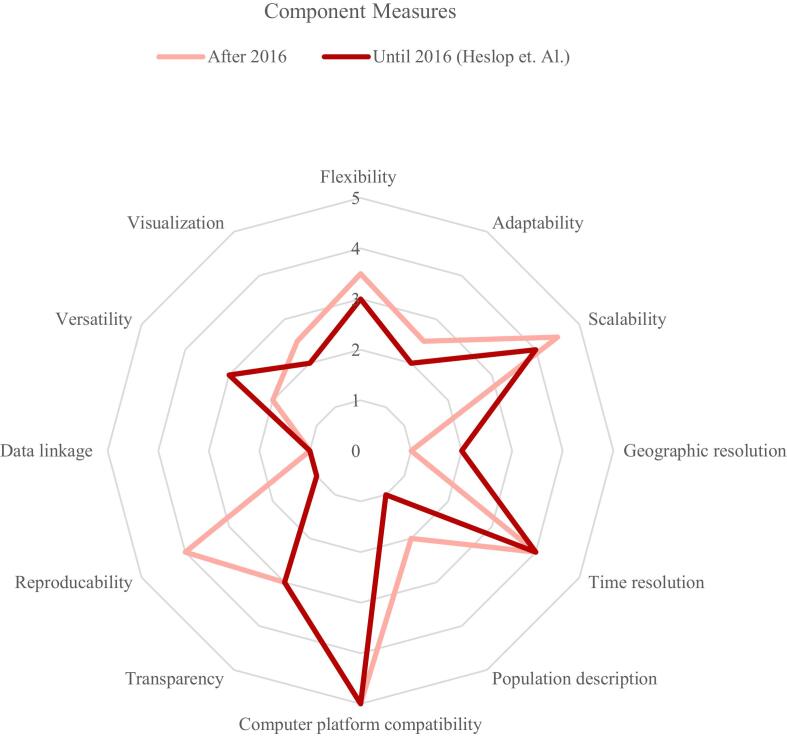
Component measurement comparison.

The updated search strategy identified twenty-three tools developed after July 2016. From the twenty tools analyzed in the 2017 review by Heslop et al., only six were re-evaluated due to others being non-functional or inaccessible. The Fig. 3 displays the component measure ratings of tools developed post-2016 against those tested by Heslop et al. (2017) until July 2016. Among the six pre-2016 tools re-assessed, four were rated below the median assessment threshold of 60 %.

These four tools also demonstrated limitations in adaptability and, in particular, reproducibility, scoring lower in utility while receiving moderate to high usability scores. Conversely, two tools, GER and GleamViz, were rated above the median. GleamViz, notable for its extensive feature set, achieved moderate component measure ratings and a favorable utility score but lower usability ratings.

## 4 Discussion

COVID-19 has significantly heightened attention on epidemiological modeling and has influenced global decision-making [31]. Building upon a previous systematic review conducted by Heslop et al. [1], this scoping review evaluates open-source software tools designed for pandemic and epidemic planning, preparation, and response developed after July-2016. The findings offer an overview of existing tools and assessment of their components, features, utility, and usability. This enables decision-makers to quickly identify the most suitable tools for their needs while also becoming aware of other available options. Such information is particularly valuable in rapid decision-making processes, enriches their knowledge base and serves as a basis for training with specific tools. For example, during the COVID-19 pandemic, the spread of the disease was regularly calculated by various experts. Using a tool for additional calculations could have provided crucial support, which would have enhanced not only decision-making, but also communication, especially if the visualization features of the tools had been used. Therefore, depending on their functionality, these tools can significantly aid in making well-informed decisions. The qualitative evaluation reveals distinct differences across various dimensions, suggesting that no single tool is universally superior Specific requirements of epidemiological analysis or planned public health intervention should guide the choice of tool, as its effectiveness can greatly depend on the intended use. For instance, Covidscreen received the highest ratings in usability and utility due to its ease of use, but it offers only a limited set of features. While this may be sufficient for some use cases, it might not meet the needs of others. On the other hand, for those seeking a tool with a broader range of features that also scores high in usability and utility, GleamViz might be the better choice. Overall, the tools provide a good qualitative baseline. However, there is room for improvement in incorporating external data sources, providing detailed population descriptions, and enhancing geographic resolution to better represent disease spread.

Investigation showed that high utility can coexist with high usability and a reasonable number of features. Usability varied across the tools' platform designs, stating that good usability depends on thoughtful design rather than the type of platform (PDF, MS Excel®, software applications, or web apps). Comparing with tools analyzed by Heslop et al. [1] revealed that pre-July-2016 tools were primarily inaccessible or outdated, while accessible ones had lower utility scores. Four out of six tools also had lower usability scores, indicating that rising understanding of user experience and interface design [32] influences epidemiological tool design.

Most developed tools were web-based, offering a rapid starting process, and meeting the needs of decision-makers. However, most of the tools require a grasp of epidemiological modeling and its parameters. Decision-makers' direct use without prior training raises doubts, as highlighted by a decision-maker within the German Federal Ministry of Health. During outbreaks, the lack of familiarity with new programs poses challenges. Tailored training sessions, scalable from local to federal levels could help, but training for specific diseases like “disease X" might be impractical due to tool limitations. This underscores the necessity for tools capable of modeling various pathogens or, ideally, those sharing similar infection pathways. Even then, using a generalized tool still involves uncertainty, as epidemiological modeling inherently entails uncertainty [33].

Communicating uncertainty is crucial [34], given the potential harm if misused. Setting performance standards to ensure reliability and safety, akin to those employed for safety-critical systems, could be considered [35]. This is particularly crucial regarding the significance of software design and code quality of the simulator, especially their suitability for inspection and testing [36].

Cognitive biases in expert opinions [37] could be mitigated using these tools to introduce a range of competing and alternative conclusions and hypotheses [38]. Transitioning from scientific advice and evidence to policy decisions requires policymakers' consideration of assumptions, risk tolerance, and trade-offs, which are often complex and not easily subjected to rigorous scientific scrutiny [39]. These tools could aid in facilitating this transition.

Researchers with a baseline knowledge of epidemic modeling, neither modeling experts nor decision-makers themselves, conducted all tools' usability and utility ratings. Hence, the utility results might differ if tested by decision-makers. A purely qualitative research approach, where decision-makers test a selection of tools followed by interviews, might provide additional helpful insights into utility, usability, and component needs from decision-makers perspectives. Furthermore, assessing the tools excluded because of the coding knowledge required and then comparing these with the rating of the tools tested in this review could lead to insights on whether user-friendliness hinders quality.

Qualitative methodology and rating scales introduce response bias [40]. Despite the researchers' attempts to reduce response bias by involving impartial researchers with third-party arbitration, a degree of bias is still involved. A moderate level of consensus, with no more than a two-point difference on all measures across all tools for each qualitative assessment point, was observed among assessors. Additionally, while creating the search string, feasibility criteria had to be considered. Hence, systematic reviews were excluded to reduce the number of results, even though a snowball approach with existing systematic reviews might have been insightful. Similarly, tools requiring a paid subscription for full evaluation were excluded from this study due to a lack of funding. Since these tools are paid, different resources might have been available for their development in comparison to open-sourced tools, which may result in significant differences in usability or utility, which could affect the results. Thus, reviewing these paid tools could provide additional insights. Furthermore, authors did not compare the tools' performance regarding repeated operations utilizing identical datasets for the same pathogen. Combining this with a retrospective comparison of outcomes could be beneficial in future research to assess the accuracy and reliability of the tools. Also, while the tools were tested on Windows and Android systems, testing them under various conditions and integrating both quantitative and qualitative measures to assess usability and utility over an extended period and comparing them to emerging standards in modeling and simulation could be highly beneficial, as Heslop et al. [1] already stated.

In conclusion, these tools can support outbreak response by generating visuals, data and act as bridges between research and policymaking. None of these tools should be relied upon as the sole source of information; their results should always be verified with field experts. However, they can support, challenge, and contextualize findings, ensuring that decisions are based on the best available information, while being relatively easy to use. While user-friendly tools are available, tailored usage to specific needs is essential. Tools should be regularly updated and reviewed by experts, transparently communicate uncertainties, and incorporate insights from previous tool developers.

A clear and accessible introduction to the validation methods and estimations used in the model would greatly assist in navigating uncertainties. While many tools already feature well-designed user interfaces, this highlights the importance of consulting with user interface and user experience experts during development to enhance overall usability.

However, it's essential to acknowledge that, regardless of how well-thought-out and controlled epidemiological modeling is conducted, as Neil Ferguson stated, models are “simplified representations of reality. Models are not crystal balls” [31].

## Funding

This research received no specific grant from any funding agency in the public, commercial, or not-for-profit sectors.

## Ethics approval and consent to participate

Review articles do not require ethical approval.

## Consent for publication

Not applicable.

## CRediT authorship contribution statement

**Rebecca Sophia Lais:** Writing – review & editing, Writing – original draft, Visualization, Validation, Software, Resources, Project administration, Methodology, Investigation, Formal analysis, Data curation, Conceptualization. **Julia Fitzner:** Writing – review & editing, Validation, Supervision, Resources, Methodology, Investigation, Formal analysis, Data curation, Conceptualization. **Yeon-Kyeng Lee:** Writing – review & editing, Validation, Methodology, Investigation, Formal analysis, Data curation. **Verena Struckmann:** Writing – review & editing, Writing – original draft, Visualization, Validation, Supervision, Resources, Project administration, Methodology, Formal analysis, Conceptualization.

## Declaration of Competing interest

The authors declare the following financial interests/personal relationships which may be considered as potential competing interests:

Dr. Verena Struckmann reports article publishing charges was provided by TU Berlin University. Dr. Verena Struckmann reports a relationship with TU Berlin University that includes: employment. If there are other authors, they declare that they have no known competing financial interests or personal relationships that could have appeared to influence the work reported in this paper.
