# Supplementary material for: Open-sourced modeling and simulating tools for decision-makers during an emerging pandemic or epidemic – Systematic evaluation of utility and usability: A scoping review update
Source: Dialogues Health. 2024 Sep 3;5:100189. doi: 10.1016/j.dialog.2024.100189 (PMC11424802; doi:10.1016/j.dialog.2024.100189)
Supplement: Supplementary material [file mmc1.docx]

# **Appendix**

# **A1: KDCA expert consultation results**

| **CDC Health Economics and Modeling Unit** | | |  |
| --- | --- | --- | --- |
| **Purpose** | **Name** | **Published/ updated** | **Article/ manual** |
| Pandemic Preparedness and Response | COVID-19 Surge | 2023 | https://jamanetwork.com/journals/jamanetworkopen/fullarticle/2790518?widget=personalizedcontent&previousarticle=2790074" |
|  | COVIDTracer and COVIDTracer Advanced | 2022 | https://www.cdc.gov/coronavirus/2019-ncov/php/contact-tracing/COVIDTracerTools.htm |
|  | COVID-Vac: A tool for planning COVID-19 vaccination clinics and mass vaccination sites | 2021 | https://www.cdc.gov/vaccines/covid-19/downloads/COVID-Vac-user-manual.pdf |
| Biological Threat Planning | Maxi-Vac 2.0 | 2021 | Maxi-Vac-2.0.xlsm (live.com) |
|  | Maxi-Vac Alternative 2.0 | 2021 | https://www.cdc.gov/smallpox/bioterrorism-response-planning/max-vac/files/Maxi-Vac-Alternative-2.0.xlsm |
|  | Anthrax Assist | 2017 | Modeling Tool for Decision Support during Early Days of an Anthrax Event - Volume 23, Number 1—January 2017 - Emerging Infectious Diseases journal - CDC |
| Vaccination Planning & Evaluation | RabiesEcon | 2018 | Transmission dynamics and economics of rabies control in dogs and humans in an African city - PubMed (nih.gov) |
|  | RSV Immunization Impact Model | 2020 | https://www.sciencedirect.com/science/article/pii/S0264410X19313866 |
| Assess Public Health Surveillance | Shigellosis Tool | 2019 | https://bmcinfectdis.biomedcentral.com/articles/10.1186/s12879-019-3796-7 |
|  | PHL Impact Tool Spreadsheet [XLSM – 532 KB] | 2020 | https://www.cdc.gov/ncezid/dpei/hemu/phl-impact.html |

# **A2: Open science framework - protocol**

**Registration DOI : https://doi.org/10.17605/OSF.IO/62J5Z**

**Type of review**

A scoping review following a qualitative assessment

**Review stages**

Search, Screening, Extraction, Synthesis

**Current review stage**

finished screening

Start date

15.09.2023

End date

01.02.2024

**Background**

Public Health Emergencies can easily affect the whole world, as COVID-19 just reminded us. This consequently brought intense focus towards the importance of research, commonly used epidemiological tools for surveillance and response, the resilience of healthcare systems, and the need for fast decision-making within policy and healthcare. Learning from such an outbreak and being prepared for what is to come next, is of the highest importance, considering that emerging infectious diseases will continue to represent a challenging global threat (Hao et al. 2022) in today's interconnected world (McArthur 2019). Epidemiological modeling can support decision-making throughout Pandemics, as just seen throughout the COVID-19 and the influenza pandemic (Adiga et al. 2020; Vanagas et al. 2019). They enable the provision of quickly generated guidance (Zaman et al. 2017; Nokes and Anderson 1988) and consolidating scientific knowledge about various factors relevant to decision-making, which would be difficult or impossible to measure empirically, such as long-term consequences of policy alternatives (James et al., 2021; (Pagel and Yates 2022). Researchers trained in various software languages and tools can program epidemic models based on different equations to simulate and analyze pandemic and epidemic dynamics (Deng et al. 2022). However, the connection and translation from modelers to decision-makers and health system managers is often lacking, which leads to results which might not be understandable for decision-makers and health system managers (Morgan 2019). Nevertheless, decision-makers facing outbreaks urgently need to understand and benefit from modeling and simulation. Hence, bridging the gap between academic requirements and practical decision-making is crucial (Yu et al. 2021). Open-sourced software tools for modeling or simulation can contribute to such a bridge but may be simplified or difficult to use (Heslop et al., 2017). Additionally, it is essential to have a closer look at the framework of a model, as modeling is only useful if used correctly (Panovska-Griffiths et al. 2021). For these tools to be utilized, decision-makers need to be aware of their existence while access and usage should be easy. Published by Heslop et al. (2017), the review "Publicly available software tools for decision-makers during an emergent epidemic—Systematic evaluation of utility and usability" provides an overview of usable tools available until July 2016. However, especially considering the intense use of modeling during the COVID-19 pandemic (Dangerfield et al. 2023), more modeling tools might have been created, and existing ones have most likely been updated. This scoping review aims to update the review by Heslop et al. (2017) to identify and list open-sourced modeling tools created after July 2016, re-evaluate the tools described in the existing review and evaluate new ones, to see whether the tools are open-sourced or openly available, assess what minimal data set has been used and which equation is used to model.

**Primary research question(s)**

What stand-alone software tools are open-source available for simulating or modeling in case of emerging pandemics or epidemics, specifically designed for decision makers and public health practitioners?

**Secondary research question(s)**

What frameworks are utilized for modeling, and what are their limitations and capabilities? How useful and user-friendly are these tools, and are they open-sourced or freely available? What minimal data set was used in each used model?

**Expectations / hypotheses**

H1: Over the past 7 years more modeling tools were developed H0: Over the past 7 years, not many new modeling tools were developed.

**Dependent variable(s) / outcome(s) / main variables**

Are the tools:

open-source or openly available

- usability
- utility
- Features
- Framework

**Independent variable(s) / intervention(s) / treatment(s)**

- Flexibility
- Adaptability
- Scalability
- Geographic Resolution
- Time Resolution
- Population Resolution
- Computer Platform Compatibility

**Additional variable(s) / covariate(s)**

- additional functions - support systems - Data in- and output functions

**Software**

- Citavi 6
- MS Excel® Version 2208
- Rayyan — a web and mobile app for systematic reviews
- Google Sheets

**Funding**

This work does not receive any funding.

Conflicts of interest

There are no conflicts of interest.

Overlapping authorships

There is no conflict expected, as the co-authors published in fields clearly different from the current work.

**Search Strategy**

In this section, you register your search strategy: the procedures you designed to obtain all (potentially) relevant sources to review (e.g., articles, books, preprints, reports, case law, policy papers, archived documents).

**Databases**

Peer reviewed databases: - Pubmed - Embase - Medline - Cochrane

Interfaces

- PubMed via Ovid - Embase via Ovid - Medline via Ovid - Cochrane via Cochrane

Grey literature

- Yahoo, Google, Bing - WHO Iris, CDC, - Base, Opengrey

**Inclusion and exclusion criteria**

Inclusion criteria:

- The tool is for epidemiological modeling or simulation of infectious diseases with pandemic or epidemic potential
- It is open sourced or at least openly available with complete usage possibility
- The tool can be completely tested in its free version and is working
- All tools assessed by Heslop et al 2017

Exclusion criteria:

- Tool requires a payment or license for its full use
- Tools which are not working (installation problems, malfunction)
- Tools without legal copyright
- Tools which require an additional software like “R”
- Systematic review

**Query strings**

1 Software/ or (Software* or tool*).ab,ti. 2 (((Epidemics/ or Pandemics/ or Zoonoses/ or Communicable Diseases/ or Disease Outbreaks/ or Infectious Disease Transmission, Vertical/ or Disaster Planning/ or ("epidemic$1" or "pandemic$1" or "preparedness" or "outbreak*" or "state-of-emergency" or "public-emergenc*" or "international-concern*" or "PHEIC" or "public-health-emergenc*" or "public-emergenc*").ab,ti. or ("infectious adj2 disease adj2 transmission$1" or "disease adj2 spread" or "infectious diseas*" or "communicable disease$1").ab,ti.) not Neoplasms/) or HIV/) not ("non-communicable disease*" or "NCD*" or "non communicable disease*" or "chronic disease*" or "cancer" or "HIV").ab,ti. 3 (simulat* or "computer-based adj1 simulat*" or "computer adj1 based adj2 simulat*" or "comput* tool*" or "simulat* adj2 software" or "model* adj3 tool*" or "model* software" or "scenario*").ab,ti. 4 Computer Simulation/ or Mathematical Computing/ or Decision Support Techniques/ or Epidemiological Models/ or Models, Statistical/ or Models, Theoretical/ or model*.ab,ti. 5 1 and 2 and 3 and 4 6 limit 5 to humans 7 limit 6 to ep=20160730-20230907

**Search validation procedure**

The search query was tested by adding the title of the following 'test-paper' to the search query with the boolean operator 'AND': - The COVID-19 pandemic preparedness simulation tool: CovidSIM.m_titl. - The GLEaMviz computational tool, a publicly available software to explore realistic epidemic spreading scenarios at the global scale.m_titl. All paper were included in the search query.

**Other search strategies**

One expert consultation to find relevant tools is planned.

One expert consultation on the best rated 10% of the tools.

**Procedures to contact authors**

Contacting authors is not planned.

Results of contacting authors

Contacting authors is not planned.

Search expiration and repetition

No search repetition is planned.

Search strategy justification

In order to search for grey literature, query strings for each database is being created. The queries are similar to the query string for peer reviewed databases, but as they are not alle using with boolean operators, filters or subject headings, they differ. As also online search engines like Google, Yahoo and Bing are being used, the queries differ but try to use same terms of the peer reviewed databases. The first 10 pages will be searched, using the strategy of previous scoping reviews which included online searches. Even though not all above listed databases were searched within the existing systematic review (Heslop et. al, 2017), for feasibility reasons, also these databases are being searched from 2016 - today.

Miscellaneous search strategy details

No additional description of details needed.

**Screening**

In this section, you register your screening procedure: the procedure you designed to eliminate all irrelevant sources from the results of the search strategy (and retain the relevant sources).

Screening stages

1st stage: Title and abstract screening (two researchers) 2nd stage: Deduplication (done with Rayyan, supervised by one researcher ) 3rd stage: Full text screening (two researchers)

Screened fields / blinding

Bibliographic fields visible: Title, abstract, authors, year Bibliographic fields blinded: Journal names,

Used exclusion criteria

• Tool requires a payment or license for its full use • Tools which are not working (installation problems, malfunction) • Tools without legal copyright • Tools which require an additional software like “R” • Systematic review

Screener instructions

- A complete PRISMA Protocol containing: - Introduction - Objectives -Eligibility Criteria - Information Sources - Exclusion and Inclusion criterias, examples for each case - Explanation of the data collection process -

*No files selected*

Screening reliability

- 2 Screeners (YL;RL) for Title and Abstract screening - 1 different screener (SKS) for conflicts - 2 Screeners for 10% of the Fulltext (YL;RL)

Screening reconciliation procedure

conflicts within screening results were dealt with by involving a third screener

Sampling and sample size

all studies identified at this stage are kept

Screening procedure justification

Peer reviewed databases: - Screened with rayyan by 2 screeners in the blind mode - Exclusion criteria were named by each screener once excluded within rayyan - Conflicts were screened by a third screener in blind mode

Data management and sharing

Search strategy and the search process is saved in an xlsx file

Miscellaneous screening details

screening of the grey literature and the online databases is done in an MS Excel® sheet. Results of search engines were copied in an MS Excel® sheet, assuring the documentation of the screened titles and prevent adjusted resultslists.

**Extraction**

In this section, you register your plans for data extraction: the procedures you designed to extract the data you are interested in from the included sources. Examples of such data are text fragments, effect sizes, study design characteristics, year of publication, characteristics of measurement instruments, final verdicts and associated penalties in a legal system, company turnovers, sample sizes, or prevalences.

Entities to extract

Qualitative data fragments: - Features of the tool - Availability - Utility and Usability - Framework of the tool Metadata: - Authors - Intitutions - Year of Publication

Extraction stages

a final extraction stage in paralell by two researchers is conducted (YL,RL) - Each tool will be tested and assessed by 2 researchers - Differences in the assessment will be supervised by a third researcher

Extractor instructions

The extractors recieve a created rating scheme

*No files selected*

Extractor masking

not applicable.

Extraction reliability

not applicable.

Extraction reconciliation procedure

a third researcher will evaluate tools with divergent rating.

Extraction procedure justification

not applicable.

Data management and sharing

Data will be shared using google drive and files in xlsx format.

Miscellaneous extraction details

not applicable.

**Synthesis and Quality Assessment**

In this section, you register the procedure for the review’s synthesis: the procedure you designed to use the data that was extracted from each source to answer your research question(s). This often includes transforming the raw extracted data, verifying validity, applying predefined inference criteria, interpreting results, and presenting results. Additionally, you register procedures you designed to assess bias in individual sources and the synthesis itself.

Planned data transformations

qualitative data fragments will be assessed according to the created rating scheme.

Missing data

Tools which cannot be assessed by the researchers are excluded.

Data validation

The rating scheme is created based on the previous done review, added with criteria based on literature and expert consultation.

Quality assessment

As this is a scoping review, there will be no assessment of the quality of the included sources.

Synthesis plan

Tools will be tested and rated according to the rating scheme. Tools which cannot be tested are excluded of the study.

Criteria for conclusions / inference criteria

conclusions are not based on pre-specified criteria

Synthesist blinding

10 % of the tools will be tested by a not involved researcher.

Synthesis reliability

2 Sythesists will be working on the synthesis.

Synthesis reconciliation procedure

An not involved third researcher will test divergent synthesis.

Publication bias analyses

not applicable.

Sensitivity analyses / robustness checks

The highest rated 10 % of the tools will be tested by not involved experts.

Synthesis procedure justification

Tools will be rated in seperate MS Excel® sheets without access to the other researchers.

Synthesis data management and sharing

Google drive is used for data sharing.

Miscellaneous synthesis details

not applicable.

# **A3: Search strings**

A3.1 Search engines: Same search string used in Google, Yahoo, Bing

| **Search No.** | **Keywords** |
| --- | --- |
| 1 | Epidemiological modeling tool |
| 2 | Epidemiological modeling software |
| 3 | Epidemiological modeling software for decision making |
| 4 | Disease outbreak modeling tool |

A3.2 Peer-reviewed data bases: created in Medline and then adapted to Embase, PubMed, Cochrane

| **Medline** | |
| --- | --- |
| **Search** | **Terms** |
| 1 | Software/ or (Software* or tool*).ab,ti. |
| 2 | (((Epidemics/ or Pandemics/ or Zoonoses/ or Communicable Diseases/ or Disease Outbreaks/ or Infectious Disease Transmission, Vertical/ or Disaster Planning/ or ("epidemic$1" or "pandemic$1" or "preparedness" or "outbreak*" or "state-of-emergency" or "public-emergenc*" or "international-concern*" or "PHEIC" or "public-health-emergenc*" or "public-emergenc*").ab,ti. or ("infectious adj2 disease adj2 transmission$1" or "disease adj2 spread" or "infectious diseas*" or "communicable disease$1").ab,ti.) not Neoplasms/) or HIV/) not ("non-communicable disease*" or "NCD*" or "non communicable disease*" or "chronic disease*" or "cancer" or "HIV").ab,ti. |
| 3 | (simulat* or "computer-based adj1 simulat*" or "computer adj1 based adj2 simulat*" or "comput* tool*" or "simulat* adj2 software" or "model* adj3 tool*" or "model* software" or "scenario*").ab,ti. |
| 4 | Computer Simulation/ or Mathematical Computing/ or Decision Support Techniques/ or Epidemiological Models/ or Models, Statistical/ or Models, Theoretical/ or model*.ab,ti. |
| 5 | 1 and 2 and 3 and 4 |
| 6 | limit 5 to humans |
| 7 | limit 6 to ep=20160730-20230907 |

A3.3 Grey Literature

| **OpenGrey** | |
| --- | --- |
| Filter | Terms |
| Audience - Life,sciences, medicine and health care | (software* OR tool* ) AND (Disease Transmission OR Disease Outbreaks OR epidemic* OR pandemic* OR Zoonoses OR Communicable diseases OR communicable diseases, emerging OR Disaster Planning OR preparedness OR outbreak* OR state-of-emergency OR public-emergenc* OR international-concern* OR PHEIC OR public-health-emergenc* OR public-emergenc* ) AND **(simulat* OR computer-based simulat* OR computer simulation OR software simulat* OR model tool OR model software OR scenario*) AND (Mathematical Computing OR Decision Support Techniques OR Epidemiological Models OR Model* OR Computer Simulation OR computational tool*) NOT** (Neoplasms OR Noncommunicable Disease* OR chronic disease* OR in-vitro OR HIV OR NCD OR chronic disease* OR cancer) |
|  |  |
| **Base** | |
| Terms | |
| (Software tool) AND (epidemic pandemic preparedness outbreak "state-of-emergency" "public emergency" "international concern" PHEIC "publi-health-emergency" "infectious disease transmission" "disease spread" "infectious disease" "comminicable disease") year:[2016 TO 2023] | |

A3.4 Stakeholder websites: CDC and WHO IRIS

| **CDC EID – Advanced article search** | |
| --- | --- |
| Combinded with OR |  |
| **Field** | **Keyword** |
| Abstract | Software |
| Abstract | Tool |
| Abstract | Simulation |
| Abstract | Computer simulation |
| Abstract | scenario |
| Abstract | decision support models |
| Keyword | decision making |
| Keyword | decision support, techniques, models, economic, perspective |
| Keyword | disease modeling |
| Keyword | statisitacl modeling |
| Abstract | modeling |
| Keyword | epidemiological modeling |
| Keyword | epidemiological models |

| **WHO IRIS** |
| --- |
| **search I** |
| epidemiological modelling [All of IRIS] AND English [Language] AND 2016 [date issued] AND tools [title] |
| epidemiological modelling [All of IRIS] AND English [Language] AND 2017 [date issued] AND tools [title] |
| epidemiological modelling [All of IRIS] AND English [Language] AND 2018 [date issued] AND tools [title] |
| epidemiological modelling [All of IRIS] AND English [Language] AND 2019 [date issued] AND tools [title] |
| epidemiological modelling [All of IRIS] AND English [Language] AND 2020 [date issued] AND tools [title] |
| epidemiological modelling [All of IRIS] AND English [Language] AND 2021 [date issued] AND tools [title] |
| epidemiological modelling [All of IRIS] AND English [Language] AND 2022 [date issued] AND tools [title] |
| epidemiological modelling [All of IRIS] AND English [Language] AND 2023 [date issued] AND tools [title] |
|  |
| **Search II** |
| model [All of IRIS] AND English [Language] AND communicable disease [MeSH] NOT chronic disease [MeSH] NOT cancer [MeSH] NOT HIV [MeSH] NOT non communicable disease [MeSH] NOT NCD [MeSH] AND Epidem [MeSH] AND limit to 2016-2023 |

| **A4 : A****ccess links of tested tools** | | | |
| --- | --- | --- | --- |
| **Name** | **Abbreviation** | **Article** | **Link** |
| A dynamic modeling tool | - | https://www.ncbi.nlm.nih.gov/pmc/articles/PMC7229979/ | spreadsheet based |
| Anthrax Assist | - | Modeling Tool for Decision Support during Early Days of an Anthrax Event - PMC (nih.gov) | https://view.officeapps.live.com/op/view.aspx?src=https%3A%2F%2Fwww.cdc.gov%2Fsmallpox%2Fbioterrorism-response-planning%2Fmax-vac%2Ffiles%2FMaxi-Vac-Alternative-2.0.xlsm&wdOrigin=BROWSELINK |
| Columbia Prediction of Infectious Diseases | CPID | - | https://cpid.iri.columbia.edu/ |
| Coronavirus Policy Response Simulator | - | - | https://budgetmodel.wharton.upenn.edu/issues/2020/5/1/coronavirus-reopening-simulator |
| CoSim | - | Viruses \| Free Full-Text \| Model-Based Analysis of SARS-CoV-2 Infections, Hospitalization and Outcome in Germany, the Federal States and Districts (mdpi.com) | https://shiny.covid-simulator.com/covidsim/ |
| Covid Web app | - | A versatile web app for identifying the drivers of COVID-19 epidemics \| Journal of Translational Medicine \| Full Text (biomedcentral.com) | http://covid-webapp.numerusinc.com/ |
| Covid-19 Exposure Assessment Tool | CEAT | Covid-19 Exposure Assessment Tool (CEAT): Easy-to-use tool to quantify exposure based on airflow, group behavior, and infection prevalence in the community - PMC (nih.gov) | https://www.cov-irt.org/exposure-assessment-tool/ |
| COVID-19 Hospital Impact Model for Epidemics | CHIME | ncbi.nlm.nih.gov/pmc/articles/PMC9580422/pdf/42979_2022_Article_1441.pdf | https://penn-chime.phl.io/ |
| COVID-19Surge | - | - | https://www.cdc.gov/coronavirus/2019-ncov/hcp/COVIDSurge.html |
| Covid19Vaxplorer | - | https://www.medrxiv.org/content/10.1101/2023.06.15.23291472v1.full.pdf | https://covid19vaxplorer.fredhutch.org/ |
| Covidscreen | - | covidscreen: a web app and R Package for assessing asymptomatic COVID-19 testing strategies \| BMC Public Health \| Full Text (biomedcentral.com) | https://sjbiostat.shinyapps.io/covidscreen/ |
| CovidSim | - | The COVID-19 pandemic preparedness simulation tool: CovidSIM - PubMed (nih.gov) | http://covidsim.eu/ |
| COVIDTracer | - | Estimated COVID-19 Cases and Hospitalizations Averted by Case Investigation and Contact Tracing in the US \| Public Health \| JAMA Network Open \| JAMA Network | https://www.cdc.gov/coronavirus/2019-ncov/php/contact-tracing/COVIDTracerTools.html#anchor_1608665663535 |
| Drive Through Mass Vaccination Sim for COVID19 | - | Artificial Intelligence Model of Drive-Through Vaccination Simulation - PMC (nih.gov) | https://www.cdc.gov/coronavirus/2019-ncov/php/contact-tracing/COVIDTracerTools.html#anchor_1608665663535 |
| Epidemix | - | Epidemix \| Epi-interactive | https://models.epidemix.app/ |
| Epilocal | - | Epilocal: A real-time tool for local epidemic monitoring (Volume 44 - Article 12 \| Pages 307–332) - Demographic Research (demographic-research.org) | https://ubasellini.shinyapps.io/EPILOCAL/ |
| Epipop | - | Tools (gleamproject.org) | https://epi-pop.org/ |
| Generic Ebola response | GER |  | https://stacks.cdc.gov/view/cdc/24900 |
| GleamViz | - | GLEAMviz \| The Global Epidemic and Mobility Model | https://www.gleamproject.org/ |
| Mapping the Risk of International Infectious Disease Spread | Mriids | Using digital surveillance tools for near real-time mapping of the risk of infectious disease spread \| npj Digital Medicine (nature.com) | https://www.mriids.org/ |
| Modeling Codiv-19 epidemics | - | Modeling COVID-19 epidemics in an Excel spreadsheet to enable first-hand accurate predictions of the pandemic evolution in urban areas \| Scientific Reports (nature.com) | spreadsheet, supplementary information 1 |
| Netlogo Epidem | - | A Selection of Software Tools for Epidemiologic Modeling \| Download Table (researchgate.net) | https://ccl.northwestern.edu/netlogo/models/epiDEMBasic |
| PHL Impact Tool | - | - | https://view.officeapps.live.com/op/view.aspx?src=https%3A%2F%2Fwww.cdc.gov%2Fncezid%2Fdpei%2Fxls%2Fphl-impact-tools.xlsm&wdOrigin=BROWSELINK |
| SISpread | - | - | https://sispread.sourceforge.net/ |
| Tabby2 | - | Tabby2: a user-friendly web tool for forecasting state-level TB outcomes in the United States \| BMC Medicine \| Full Text (biomedcentral.com) | https://ppmltools.org/tabby2/ |
| Texas Pandemic Flu Excercise | - |  | https://flu.tacc.utexas.edu/ |
| The Burden of Communicable Disease in Europe toolkit | BCoDE | A Software Tool for Estimation of Burden of Infectious Diseases in Europe Using Incidence-Based Disability Adjusted Life Years - PubMed (nih.gov) | https://www.ecdc.europa.eu/en/publications-data/toolkit-application-calculate-dalys |
| VacStockPile | - | - | https://stacks.cdc.gov/view/cdc/5355 |
| Weill Cornell Bioterrorism and Epidemic Outbreak Response Mode | BERM | Weill Cornell Bioterrorism and Epidemic Outbreak Response Model (BERM) - Inputs (simfluenza.org) | https://phs.weill.cornell.edu/research-collaboration/divisions-institutes/cornell-institute-disease-disaster-preparedness/pandemic |

**A5:** **List of excluded tools**

|  |  | **Tool Name** | **Web link** | **Reason for exclusion** |
| --- | --- | --- | --- | --- |
| Heslop | 1 | SimFlu | http://lcbb.snu.ac.kr/simflu/ | Disabled access link |
|  | 2 | FluAid 2.0 | http://www.cdc.gov/flu/pandemic-resources/tools/fluaid.htm | Disabled access link - only archived not updated since review |
|  | 3 | CommunityFlu 2.0 | http://www.cdc.gov/flu/pandemic-resources/tools/communityflu.htm | Disabled access link - only archived not updated since review |
|  | 4 | StatFlu | http://www.s-gem.se/statflu/ | Disabled access link |
|  | 5 | FluSurge 2.0 | http://www.cdc.gov/flu/pandemic-resources/tools/flusurge.htm | Disabled access link - only archived not updated since review |
|  | 6 | FluWorkLoss 1.0 | http://www.cdc.gov/flu/pandemic-resources/tools/fluworkloss.htm | Disabled access link - only archived not updated since review |
|  | 7 | AsiaFlu Cap | http://www.cdprg.org/asiaflucap-simulator.php | Disabled access link; cannot be found through link |
|  | 8 | FluLabSurge 1.0 | http://www.cdc.gov/flu/pandemic-resources/tools/flulabsurge.htm | Disabled access link - only archived not updated since review |
|  | 9 | Global Epidemic and Mobility Model | http://www.gleamviz.org/simulator/ | Disabled access link |
|  | 10 | FluTE | http://www.cs.unm.edu/~dlchao/flute/#Download | Coding Skills needed |
|  | 11 | EpiGrass | https://sourceforge.net/projects/epigrass/ | Disabled access link - only archived not updated since review |
|  | 12 | Framework for Reconstructing Epidemiological Dynamics | http://fred.publichealth.pitt.edu/simulator/ | Disabled access link |
|  | 13 | Modelling for All Project | http://m.modelling4all.org/ | Disabled access link - only archived not updated since review |
|  | 14 | Epidemiological Modelling software | http://idmod.org/ | Coding skills needed |
| Found in this review | 15 | AEGIS | https://www.ncbi.nlm.nih.gov/pmc/articles/PMC7663469/ | Coding skills needed; R Package needed |
|  | 16 | AIDO | https://aido.bsvgateway.org/ | Other; Just visualization tool |
|  | 17 | Anylogic | https://www.anylogic.de/ | Payment needed |
|  | 18 | Anytown, USA | https://covidweb.isi.jhu.edu/simulator | Malfunction; page does not work |
|  | 19 | Compartmental Modeling Software (CMS) | https://github.com/InstituteforDiseaseModeling/IDM-CMS | Coding Skills needed |
|  | 20 | Sormas | https://demo.sormas.org/sormas-ui/#!dashboard/surveillance | Other; surveillance not modeling |
|  | 21 | Covasim | https://github.com/InstituteforDiseaseModeling/covasim | Coding Skills needed |
|  | 22 | Covid Sim | https://github.com/lvanhee/COVID-sim | Coding Skills needed |
|  | 23 | COVID-19 Spread Mapper | https://covid19-analysis.org/ | Malfunction |
|  | 24 | Decision support for evidence-based integration of disease control | https://journals.plos.org/plosntds/article?id=10.1371/journal.pntd.0006328#sec011 | Other; tool not found |
|  | 25 | DengueME | https://github.com/ufopleds/DengueME/wiki/How-to-Install | Coding Skills needed |
|  | 26 | multiagent modeling to forecast the spatiotemporal development of the COVID-19 pandemic | GitHub - piotrpowerpalka/Covid-19-ABM at PPbranch | Coding Skills needed |
|  | 27 | STEM Spatiotemporal Epidemiologic Modeler | https://projects.eclipse.org/projects/technology.stem | Malfunction; download not possible |
|  | 28 | TS power calculator for count data | https://www.ncbi.nlm.nih.gov/pmc/articles/PMC7578561/ | Malfunction |
|  | 29 | HelicsWin.net (HWN) | https://www.ecdc.europa.eu/en/publications-data/helicswinnet-hwn | Hospital codes needed for testing |
|  | 30 | Memilio | https://github.com/SciCompMod/memilio/releases | Coding Skills needed |
|  | 31 | Repast | https://sourceforge.net/projects/repast/ | Coding Skills needed |
|  | 32 | Multi-Country Covid-19 Projection Tool | https://unicef-my.sharepoint.com/:x:/g/personal/moserrano_unicef_org/EXhana409zVOvb7AcYrK_HABX8jVfkb2K1Bs1ZkHS2bBPw?rtime=sdTdc6H420g | Malfunction; tool cannot be tested, read only function |
|  | 33 | MIcro Simulation Tool (MIST) | https://github.com/Jacob-Barhak/MIST | Malfunction; installation not functional |
|  | 34 | Malaria-VisAnalytics | http://ipef2020.ddns.net:3838/malaria/ | Malfunction; page does not work |
|  | 35 | K-SEIR-Sim | http://peiyun.cn/download/seir_sim.files/SEIR_sim_V2.53.exe | Malfunction; download not functional |
|  | 36 | Infectious Diseases Seaker (IDS) | https://www.ncbi.nlm.nih.gov/pmc/articles/PMC8003641/#:~:text=IDS%20is%20a%20standalone%20software,without%20requiring%20a%20network%20connection. | Other; tool not found in paper, only description |

# **A6: Inclusion and exclusion criteria**

Inclusion criteria:

- The tool is for epidemiological modeling or simulation of infectious diseases with pandemic or epidemic potential
- It is open sourced or at least openly available with complete usage possibility
- The tool can be completely tested in its free version and is working
- All tools assessed by Heslop et al 2017

Exclusion criteria:

- Tool requires a payment or license for its full use
- Tools which are not working (installation problems, malfunction)
- Tools without legal copyright
- Tools which require an additional software like “R”
- Systematic review
